# Effects of cyclic equibiaxial mechanical stretch on α-BK and TRPV4 expression in equine chondrocytes

**DOI:** 10.1186/2193-1801-3-59

**Published:** 2014-01-29

**Authors:** Ismail M Hdud, Ali Mobasheri, Paul T Loughna

**Affiliations:** School of Veterinary Medicine and Science, Faculty of Medicine and Health Sciences, The University of Nottingham, Sutton Bonington Campus, Leicestershire, LE12 5RD UK; Medical Research Council-Arthritis Research UK Centre for Musculoskeletal Ageing Research, Leicestershire, UK

**Keywords:** Chondrocyte, Mechanical stretch, TRPV4, BK channel, Mechanotransduction

## Abstract

**Background:**

Chondrocytes are regularly exposed to load-induced stimuli and have the capability to sense and respond to applied mechanical stress. However, the mechanisms involved in chondrocyte mechanotransduction are not clearly understood. The purpose of this study was to explore the effects of cyclic equibiaxial mechanical stretch on the expression of α-BK and TRPV4 channels.

**Findings:**

Freshly isolated equine articular chondrocytes were subjected to mechanical stress (8% elongation at frequency of 0.5 Hz for 8 h). Western blotting was used to investigate the expression of BK_Ca_ and TRPV4 channel proteins. Mechanical stretch increased the expression of BK_Ca_ channels by 1.8 fold but TRPV4 expression was not affected.

**Conclusions:**

Upregulation of BK_Ca_ channel may be the result of direct membrane stretch or elevated intracellular Ca^2+^.

## Introduction

Chondrocytes are the only resident cells within the extracellular matrix (ECM) of articular cartilage (Archer and Francis-West, 2003). They are highly sensitive to mechanical load and are routinely exposed to a diverse variety of mechanical stimuli (Urban, 1994). Although biomechanical factors are important for articular chondrocyte metabolism and the synthesis, maintenance and degradation of ECM (Inoue et al., 1990) excessive or inappropriate mechanical loads can lead to harmful effects on cartilage. This in turn can lead to the initiation and progression of joint diseases such as osteoarthritis (OA) (Guilak, 2011).

Stretch-induced deformation of the chondrocyte membrane is thought to be one of the key processes involved in the responses to mechanical stimulation (Martina et al., 1997). The ion channels involved in chondrocyte mechanotransduction pathways have not been unambiguously identified. However, there are several candidates including large conductance “big” potassium channels (BK) and transient receptor potential vanilloid (TRPV) channels. BK channels are activated by increases in [Ca^2+^]_i_, and membrane potential (Huang et al., 2011). BK channels play an important role in various physiological functions such as regulation of vascular smooth muscle tone, endocrine cell secretion and neuronal firing (Xiang et al., 2011). Recent studies have identified BK channels in articular chondrocytes (Mobasheri et al., 2010). TRPV channels, especially isoforms 1–4 are moderately Ca^2+^ permeable channels (Phan et al., 2009; Nilius and Owsianik 2011) that play diverse roles in cellular function and TRPV4 seems to have a mechanosensory role (Wang et al., 2011, Liedtke et al., 2000). The aim of this study was to determine whether the expression levels of these purported mechanosensory ion channels are themselves modulated by mechanical signals.

## Methods

Cartilage shavings (including superficial and middle zones) were dissected from metacarpophalangeal joints of healthy mature horses euthanized for unrelated clinical reasons. Articular chondrocytes were isolated as previously described (Mobasheri et al., 2010). Chondrocytes were cultivated in monolayer in DMEM supplemented with 10% fetal calve serum (FCS) and 2% antibiotics. The chondrocyte phenotype was maintained by not passaging the cells beyond passage 2.

Equine articular chondrocytes were cultivated in Bioflex® tissue culture plates pre-coated with collage type I (Flexcell International Corporation USA) at 2 × 10^-5^ per well in DMEM medium and cultured at 37°C, 95% air and 5% CO_2_ until sub-confluent. Equibiaxial mechanical stretching was conducted using an *in vitro* system (Flexercell Strain Unit, FX-4000, Flexcell International Corporation USA) as we have described previously (Atherton et al., 2009). Medium was refreshed every other day. In the current study, chondrocytes were subjected to 8% elongation at 0.5 Hz frequency continuously for 8 hours inside the cell culture incubator. The control cells were grown on the same type of plates and kept at the same conditions without exposing to mechanical stress. Western blotting was carried out as previously described (Atherton et al., 2009) using rabbit polyclonal antibodies against TRPV4 (Abcam) and α-BK (Sigma Aldrich).

Statistical analysis: Average data are presented as a mean ± S.E.M, Student’s t-test or ANOVA was used to determine the statistical significance, followed by Bonferroni's test for multiple comparisons. *P* ≤ 0.05 considered statistically significant.

## Results and discussion

**Figure 1:**
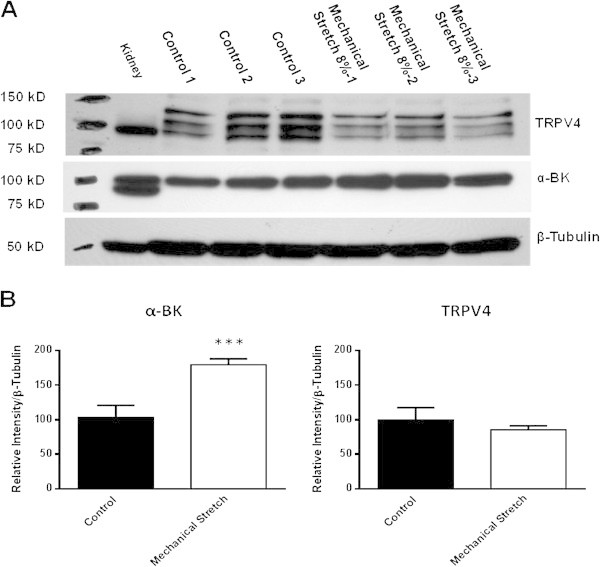
**The effects of mechanical stretch on α-BK and TRPV4 protein levels in chondrocytes. (A)** Expression of α-BK and TRPV4 channels in equine articular chondrocytes following exposure to mechanical stretch for 8 h. **(B)** The fold increase in the expression of α-BK and TRPV4 channels based on densitometric analysis of western blot by comparing to a housekeeping protein (β-tubulin). Data presented the mean ± S.E. and statistical significance is indicated by *** = *P* < 0.001 compared to unstretched controls.

Cyclic stretching significantly increased the expression of the α-BK channel (by 1.8 fold) but in contrast the expression of the TRPV4 channel was not effected by mechanical stretch (Figure 1).

The response of the α-BK channel to mechanical stretch suggests that membrane stretching may upregulate this channel directly. The α-BK channel present in articular chondrocytes may function as a mechanosensor and elevation in tensile stress could lead to activation of the channel. In the current study the increased expression of this channel was observed in response to stretch suggests that upregulated expression may be linked to the activation of the channel. Application of cyclic tensile stretch to rat growth plate chondrocytes was shown to enhance the synthesis of ECM proteins such as of collagen and aggrecan (Mouw et al., 2007).

In load bearing joints chondrocytes are exposed to mechanical forces that lead to membrane deformation, which in turn, may significantly alter cartilage matrix production (Urban, 1994). Chondrocyte membrane stretch could occur by application of mechanical compression leading to cartilage deformation and consequently, deforming the chondrocyte to an approximate elliptical shape (Liappis et al., 2011; Urban, 1994). In this study, the stretching of the membrane occurs through the cell deformation applied by direct mechanical stretch which could lead to activation of several intracellular signaling cascades. Increased expression of the BK channel through mechanical stretching could indicate enhanced responsiveness of a mechano-sensing channel (Ca^2+^-independent). An alternative hypothesis for BK channel upregulation requires the elevation of intracellular Ca^2+^ concentration (Ca^2+^-dependent) that could occur through intracellular Ca^2+^ release (Grandolfo et al., 1998) or Ca^2+^ entry through Ca^2+^ channels such as L-type voltage gated Ca^2+^ channel (VGCC) and/or members of TRP channel family. The level of TRPV4 protein was not significantly effected following exposure to mechanical stress which is interesting as it has been previously shown that the overall activity of this channel is directly related to its expression level (Veys et al., 2012).

## Conclusions

The results of this study suggest that in equine articular chondrocytes the α-BK but not the TRPV4 channel is upregulated by cyclic equibiaxial membrane stretch.
